# Comparison of a traditional systematic review approach with review-of-reviews and semi-automation as strategies to update the evidence

**DOI:** 10.1186/s13643-020-01450-2

**Published:** 2020-10-19

**Authors:** Shivani M. Reddy, Sheila Patel, Meghan Weyrich, Joshua Fenton, Meera Viswanathan

**Affiliations:** 1 RTI International, 307 Waverly Oaks Road, Suite 101, Waltham, MA 02452 USA; 2 RTI International, 3040 East Cornwallis Road, Research Triangle Park, NC 27709 USA; 3 UC Davis, Center for Healthcare Policy and Research, 2103 Stockton Blvd., Sacramento, CA 95817 USA

## Abstract

**Background:**

The exponential growth of the biomedical literature necessitates investigating strategies to reduce systematic reviewer burden while maintaining the high standards of systematic review validity and comprehensiveness.

**Methods:**

We compared the traditional systematic review screening process with (1) a review-of-reviews (ROR) screening approach and (2) a semi-automation screening approach using two publicly available tools (RobotAnalyst and AbstrackR) and different types of training sets (randomly selected citations subjected to dual-review at the title-abstract stage, highly curated citations dually reviewed at the full-text stage, and a combination of the two). We evaluated performance measures of sensitivity, specificity, missed citations, and workload burden

**Results:**

The ROR approach for treatments of early-stage prostate cancer had a poor sensitivity (0.54) and studies missed by the ROR approach tended to be of head-to-head comparisons of active treatments, observational studies, and outcomes of physical harms and quality of life. Title and abstract screening incorporating semi-automation only resulted in a sensitivity of 100% at high levels of reviewer burden (review of 99% of citations). A highly curated, smaller-sized, training set (*n* = 125) performed similarly to a larger training set of random citations (*n* = 938).

**Conclusion:**

Two approaches to rapidly update SRs—review-of-reviews and semi-automation—failed to demonstrate reduced workload burden while maintaining an acceptable level of sensitivity. We suggest careful evaluation of the ROR approach through comparison of inclusion criteria and targeted searches to fill evidence gaps as well as further research of semi-automation use, including more study of highly curated training sets.

## Background

Many clinical guideline and policy statements rely on a systematic evidence review (SR) that synthesizes the evidence base. SRs are labor-intensive projects due to high standards of validity and comprehensiveness. Guidance from the Institute of Medicine and the Effective Health Care (EHC) Program favor high sensitivity of literature searches and screening over specificity. All citations require dual review of abstracts and articles at each step of screening to identify and include all relevant research in the SR [1–3]. With the exponential growth of biomedical literature, a sensitive search can yield thousands of citations for dual abstract review and hundreds of articles for dual full-text review, whereas the number of articles ultimately included in the evidence review is typically much less.

In this study, we examine strategies to reduce workload burden of title and abstract screening as an adjunct investigation in parallel to conducting a traditional update SR on treatments for early-stage prostate cancer. Early-stage prostate cancer is a common but indolent disease that may remain clinically silent for a man’s lifetime; thus, the decision to treat clinically localized prostate cancers balances the risk of common treatment side effects (such as urinary incontinence or erectile dysfunction) with the less common risk of cancer progression and death [4–6]. In the current example, we performed an update search on comparative effectiveness of treatments for early-stage prostate cancer, including patient-centered outcomes. We relied on two recent SRs of treatments for early-stage prostate cancers: one focused on comparisons of active treatment to conservative management [7], and the second also included head-to-head comparisons of active treatments [8]. These reviews were conducted in 2014 and 2016, necessitating a SR update. We identified three approaches to updating SRs: the traditional search and screening method recommended by the EHC Program, a “review of reviews” (ROR) approach, and, semi-automation of abstract screening. A *traditional search approach* involves conducting searches in multiple databases, including searches of databases, grey literature sources, as well as a ROR: an approach intensive in labor and time. The *ROR approach* involves identifying and selecting SRs to either use in their entirety or to rely on for identifying relevant primary studies. Because the yield of eligible systematic reviews is smaller than comprehensive database searches, the effort requires less labor and time than a traditional approach but has the potential to miss relevant citations. *Semi-automation* screening software uses text-mining algorithms to find patterns in unstructured text and machine learning (ML) to train predictive classification algorithms to make inclusion and exclusion decisions or prioritize relevant citations in the title and abstract screening step of an SR [9]. Semi-automation tools have the potential to alleviate the burden on human reviewers by decreasing the number of citations to be screened by human reviewers, replacing one of two human reviewers, or using screening prioritization to improve screening workflow [10]. Active learning is a type of ML where the algorithm and reviewer interact: the algorithm generates a list of prioritized citations for the researcher to review rather than presenting unscreened citations in a random order; the next step of reviewer inclusion and exclusion decisions further train the predictive model [11].

In this study, we compare the traditional SR screening process with (1) a ROR screening approach and (2) a semi-automation screening approach using two publicly available tools (RobotAnalyst and AbstrackR). With respect to semi-automation approaches, we evaluate training sets composed of randomly selected citations from the traditional database search, highly curated citations identified from full-text review, and a combination of the two to examine ways reviewers can practically incorporate ML tools into the review workflow. Similarly, ML algorithms predict a probability that a citation will be included in the final SR, and we examine different thresholds for inclusion to understand the trade-off between inclusion probability thresholds and sensitivity when incorporating ML into SRs. We evaluate performance measures of sensitivity, specificity, missed citations, and workload burden to assess if either approach could maintain the sensitivity of a traditional review approach while reducing the number of full-text articles a reviewer would have to read.

## Methods

### Traditional review

We first conducted the traditional database search from 1 January 2014 to 27 November 2018, beginning a few months prior to the most recent reviews on the topic [7, 8]. We created search strings to replicate the yield and be consistent with the search criteria of the two more recent reviews related to the research. Appendix 1 lists our search strings for PubMed, Cochrane, and grey literature sources, specifically, HSRPproj, clinicaltrials.gov, and AHRQ and PCORI projects. Appendix 2 lists our inclusion and exclusion criteria. For studies identified through the database search, we dually and independently reviewed titles and abstracts against the inclusion and exclusion criteria. Articles flagged for inclusion by either reviewer then moved to full-text review. Each full-text article was then dually and independently reviewed. Consensus or third-party adjudication resolved conflicts. The results of the ROR (described below) were included in the final yield of the traditional review.

### Review of reviews approach

For the ROR, two team members independently selected SR citations from the database search for identification of primary research studies for further title and abstract screening and potential full-text review. The same team members conducted both the traditional search and the hand search to ensure greater consistency for the traditional search and ROR. The results of the ROR approach (i.e., ROR alone) were compared to the traditional review approach (i.e., database search and ROR).

### Semi-automation approach

For the semi-automation approach, we used two software programs that apply machine learning for title and abstract screening and are readily available for current use. *RobotAnalyst* is publicly available, web-based software, developed by the National Centre for Text Mining, with a growing evidence base across several topic areas [12, 13]. After reviewers screen at least 25 citations, the reviewer can train a ML algorithm to predict the probability that an unlabeled citation should be included. Each unlabeled citation is assigned an inclusion prediction probability (0 to 1.0). The review team can assign a threshold for the inclusion probability, above which citations are included and below which they are excluded, to reduce the number of citations that need to be manually reviewed. *AbstrackR* is an open-source tool for facilitating the citation screening process that has an active learning setting designated at the start of the review. After researchers screen a certain number of randomly selected citations, the active learning algorithm assigns a score to unlabeled citations analogous to the inclusion prediction generated by RobotAnalyst, and selects the next citation for the reviewer to screen based on a priority ranking table stored in the project database. AbstrackR periodically calls on the ML library to refine the ML algorithm and re-rank the unlabeled citations based on additional human screening decisions. The AbstrackR active learning system performs citation re-prioritization asynchronously from human review due to the computational cost, which could slow down the program and impede efficient expert review [14].

These two tools were chosen for their public availability. AbstrackR, specifically, is supported by the Agency for Healthcare Research and Practice, free to users, and is a familiar interface to Evidence-based Practice Centers for title-abstract screening. Our tests of each program included (1) creating training sets of dually reviewed abstracts, (2) invoking ML to predict inclusion probabilities of the unlabeled citations, and (3) comparing the semi-automated approach to a traditional review approach.

#### RobotAnalyst

**Fig. 1:**
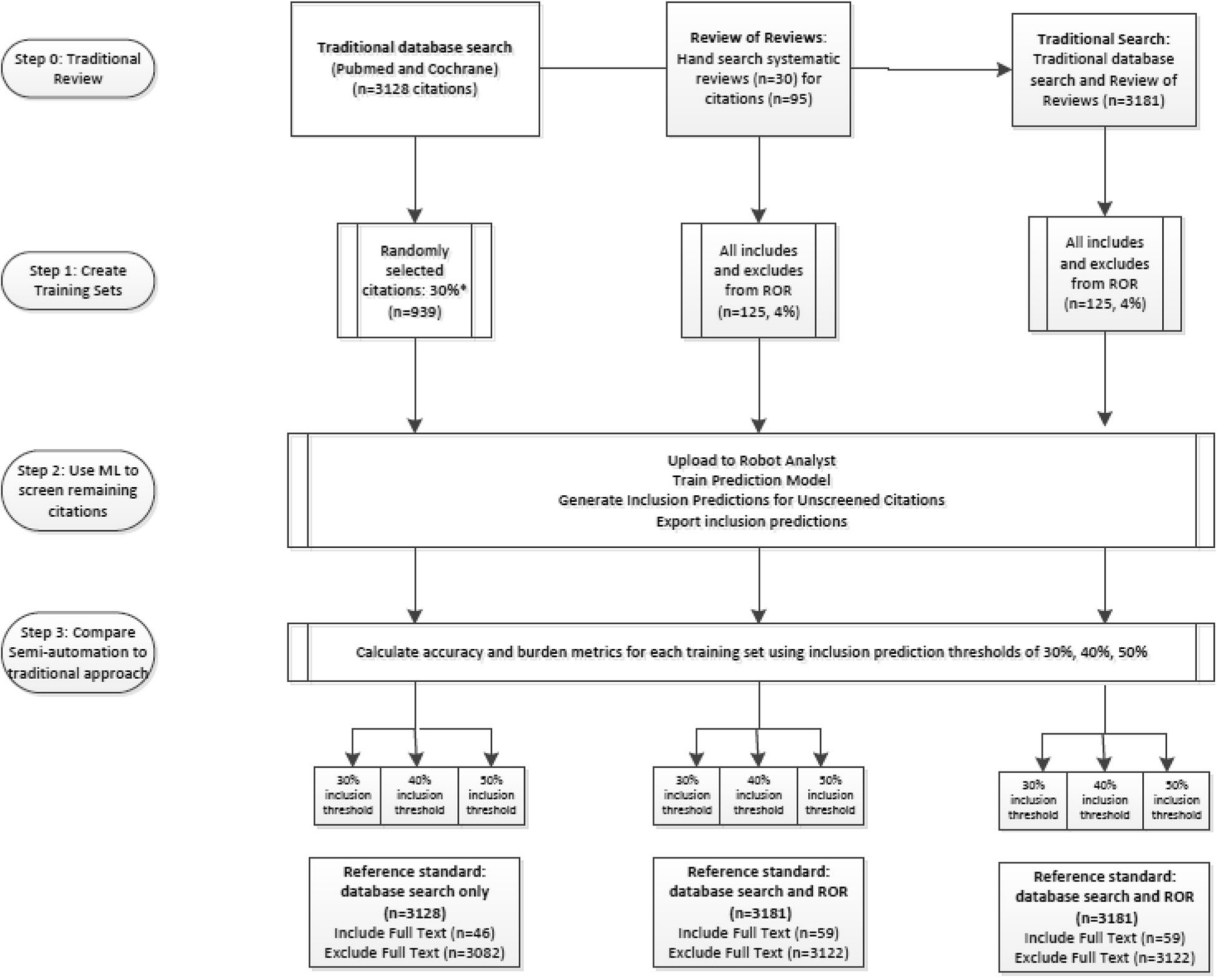
Semi-automation screening tests with RobotAnalyst

For the RobotAnalyst tests (Fig. 1), we created three training sets. We present these in order of the size and accuracy of the training set. The first training set included up to 30% of all citations retrieved from PubMed and Cochrane Library database searches. An excel random number generator was used to select 30% of dually screened citations from the traditional review (*n* = 939). Prior studies using smaller sized training sets (< 20%) have shown poor sensitivity [15, 16]. At least 5% of the citations that were included after title and abstract screening in the traditional review were included in the training set to ensure the algorithm was exposed to citations that would be included after the first stage of screening. This training set simulates prospective incorporation of ML into title-abstract screening. The training set labels are based on decisions made after dual review of title and abstracts only; thus, studies included after this first screening step may be true positives or false positives, the latter excluded after full-text review.

The second training set used inclusion decisions from the ROR (*n* = 125, 4%), specifically inclusion after full-text review. The advantage of this training set is that it contains citations that have been dually reviewed in two stages and does not include any false positives (i.e., studies screening positive at title-abstract review but negative at full-text review). This training set involves greater effort in generating the curated ROR set and simulates combining an established rapid review method with semi-automation.

The third training set combined the ROR training set and 30% randomly chosen citations with full-text decisions from the traditional review (versus title-abstract decisions). This last training set builds on the ROR training set and, as with the ROR training set, there are no false-positive labels used to train the ML algorithm. This strategy is closest in workflow to traditional systematic review processes that do not employ semi-automation, while potentially reducing the number of citations to screen by over half, though does require the greatest effort to create the training set.

The training sets were uploaded to RobotAnalyst, the reviewer explicitly called on RobotAnalyst to train the prediction model and update unlabeled citations with an inclusion prediction probability, and the probabilities were exported from RobotAnalyst for analysis. To compare a semi-automated approach to the traditional review approach, we chose three thresholds for the inclusion prediction probability: 30%, 40%, and 50%. The default setting in RobotAnalyst is 50%, and we examine two lower thresholds to assess the impact on sensitivity and reviewer burden.

#### AbstrackR

**Fig. 2:**
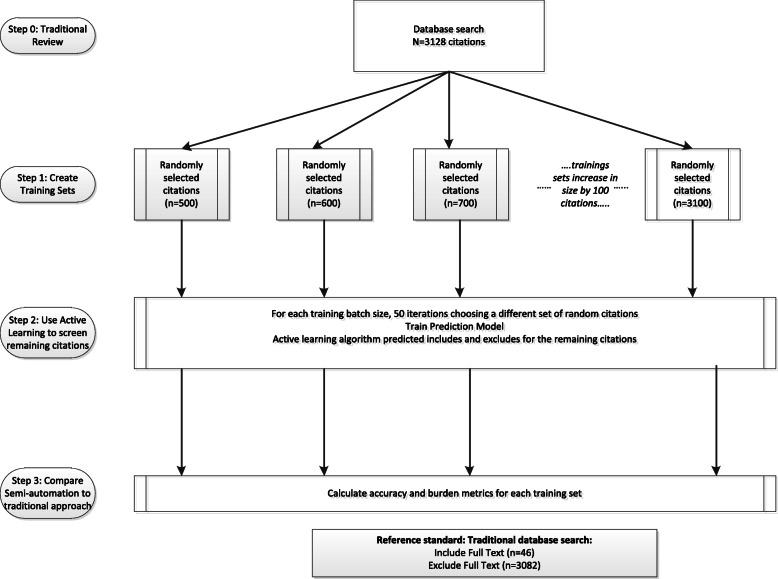
Semi-automation screening tests with AbstrackR

For the AbstrackR semi-automation test, a simulation program was used to estimate outcomes for multiple training sets, beginning with 500 randomly selected citations and ranging up to 3100 citations. (Figure 2) Because active learning used by AbstrackR is a dynamic process that occurs asynchronously from the time of screening, the initial training set of random citations and order of subsequent citations presented to the reviewer can influence final results. Thus, for each training set, 50 iterations of the simulation were performed for each training set, selecting a different set of random citations and allowing the algorithm to pick the next 50 citations that were predicted to be most likely relevant for human review. Citations with predictions falling below 0.40 were considered excludes by AbstrackR.

### Outcomes

**Fig. 3:**
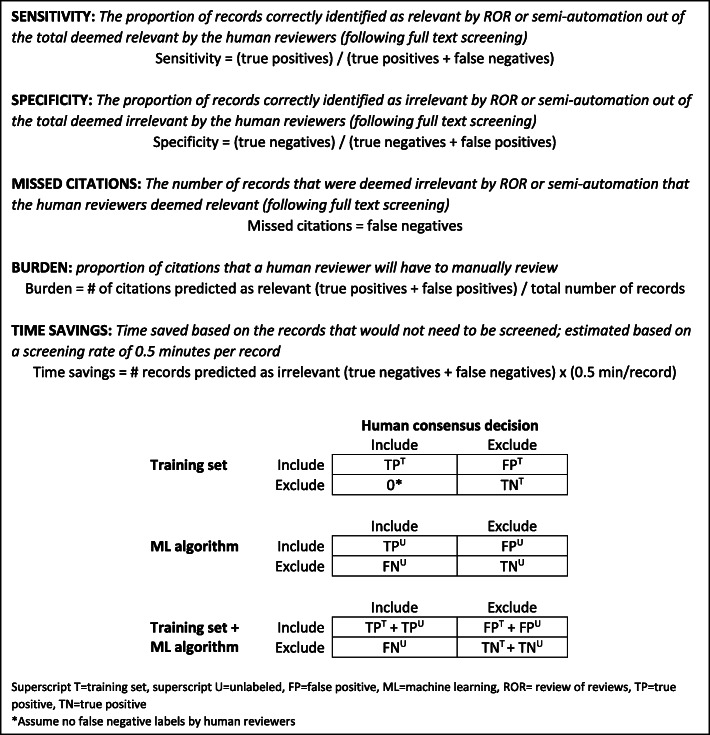
Outcome definitions and confusion matrix

Figure 3 summarizes the outcomes evaluated: sensitivity, specificity, missed citations, burden, and time saved. Time savings was calculated using an estimate of 30 s per abstract review by an experienced reviewer, as previously cited in the literature; our experience is also consistent with this estimate [16]. For the ROR approach, we further compared the inclusion criteria for the update SR with the inclusion criteria of the SRs included in the ROR to assess how well our PICOTS are represented by the ROR approach. We then describe citations missed by the ROR approach to better understand if certain study characteristics are prone to being missed by rapid review approaches.

We present results for the ROR and semi-automation approaches compared with the traditional approach. Outcomes for the semi-automation approach can be calculated for the entire process—dual human review of the training set and incorporating ML to predict inclusion of remaining citations—or for the performance of the ML algorithm alone. We present the outcomes for the entire process as our main findings because it best represents how semi-automation would be employed in current practice. (Outcomes for the ML algorithm alone are presented in Appendix 3.) Figure 3 shows the confusion matrix used to calculate outcomes incorporating the training set and ML inclusion decisions. We assumed that there were no false-negative inclusion decisions made by human reviewers. Outcomes reported for the AbstrackR semi-automation test include sensitivity and burden, also based on the entire review process as discussed above, though each outcome measure is an average of the 50 iterations performed for each of the 27 training sets and we were unable to estimate missed citations. Because AbstrackR uses active learning, we are unable to estimate outcomes for the algorithm alone, as remaining citations are not a random subset of unlabeled citations.

## Results

### Review of reviews approach

**Fig. 4:**
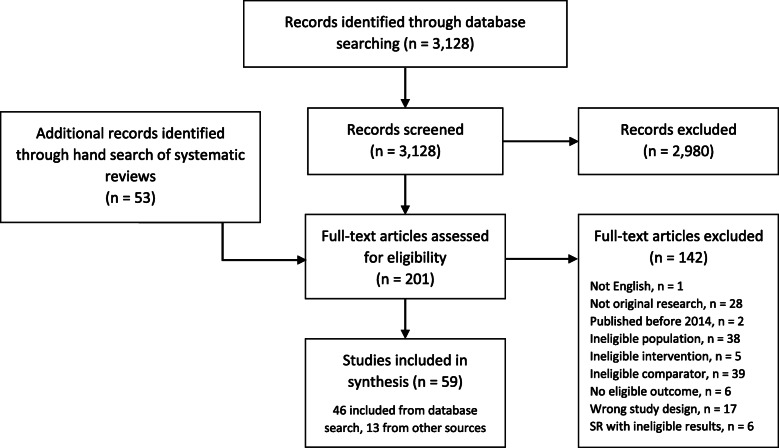
Traditional approach PRISMA

**Fig. 5:**
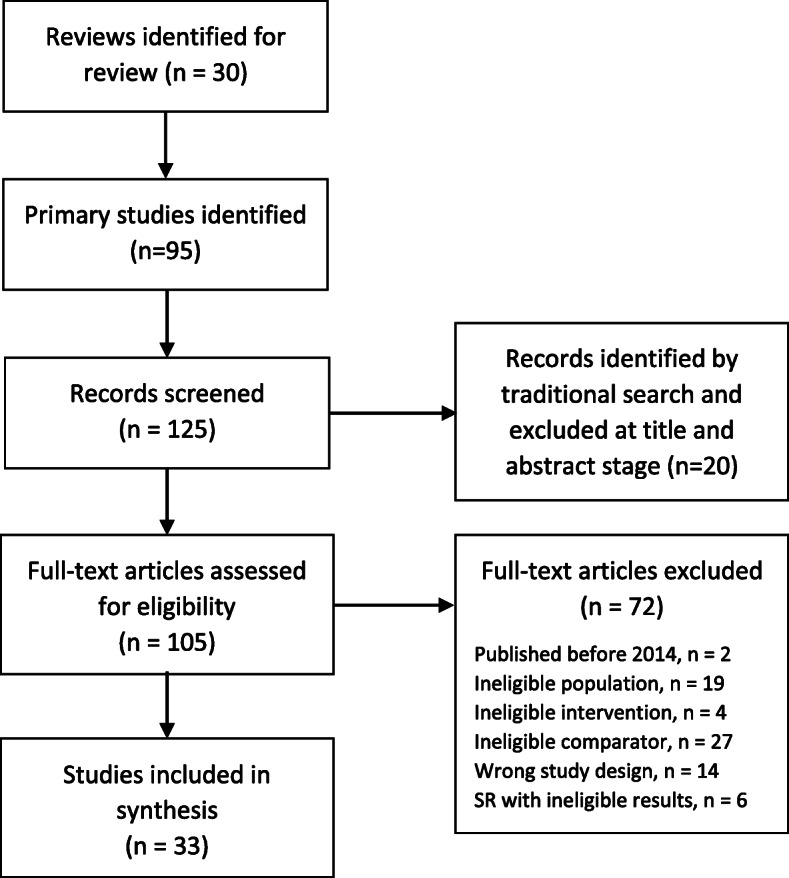
Review of reviews PRISMA

Figures 4 and 5 show the results of our traditional search and ROR. The traditional database search yielded 3128 citations, with 46 studies included after full-text screening. The ROR identified 30 systematic evidence reviews, and 95 primary studies were retrieved after a hand search. Among the 125 studies identified for screening in the ROR, 33 were included after full-text screening. The 2 × 2 matrix comparing the ROR approach to the traditional search is shown in Table 1. The ROR approach greatly reduced the number of citations screened (*n* = 125 vs. 3181, burden 4%, time savings 25.5 h), though had a low sensitivity (56%) and 26 missed citations. Table 2 summarizes the update SR inclusion criteria and overlapping inclusion criteria of SRs found in the ROR approach. Among SRs included in the ROR approach, inclusion criteria for population were well-matched to the update SR, apart from three studies that included > 10% of men with locally invasive or advanced disease. Most SRs included surgery or radiation treatment arms, active comparators, and randomized or non-randomized trials in their inclusion criteria. Ablative therapies as well as observational studies (cohorts and case control studies) and single arm study designs were less likely to be listed as SR inclusion criteria.

**Table 1:** Sensitivity and specificity of review of reviews approach compared with traditional review approach

		Traditional review approach		
		Include	Exclude	Total
**Review of reviews approach**	Include	33	0	33
	Exclude	26	3122	3148
	Total	59	3122	3181
		**Sensitivity 0.56**	**Specificity 1.00**	

*Of the 3122 excluded studies, 92 were excluded after full-text review and 3030 were citations not retrieved through the review of reviews approach

**Table 2:** Comparison of inclusion criteria for the systematic review update and systematic reviews in review of reviews (ROR) approach

	Systematic review update Inclusion criteria	SRs with inclusion criteria matching SR update eligibility criteria*	
		***N***	%
Population	Localized prostate cancer	27	90
Interventions studied in one or more arm	Active therapy (surgery and/or radiation)	17	57
	Ablative therapy only	9	30
Comparators studied in one or more arm	Active treatment comparator	17	57
	Conservative treatment comparator	11	37
	No comparator	8	27
Study design	Randomized and non-randomized trials	20	67
	Observational studies	14	47
	Single arm studies	8	27
Total		30	100

*Studies may include multiple arms or multiple study types; percentages do not sum to 100

Table 3 presents characteristics of studies missed by the ROR approach search. We observe that a significant number of missed citations are of active therapies or comparisons of active therapies. Over 50% of missed citations were published in 2016 or later (*n* = 16); among this subset of more recent studies, 11 studies had an active treatment comparator. Missed studies were much more likely to be observational studies versus trials (88 vs. 8%). Among the 25 uniquely missed studies, 16 were cohort studies (Appendix 4). The outcome of interest in 75% of the missed cohort studies were harms—quality-of-life outcomes measuring urinary or erectile dysfunction, psychological harms such as mental health disease or decision regret, and procedure—or medication-related harms.

**Table 3:** Characteristics of studies missed by the review of reviews approach

Sample size	Subtype	Studies missed by the ROR search*	
		***N***	%
Interventions studied in one or more arm	Surgery	15	60
	Radiation therapy	15	60
	Ablative therapy	10	40
Comparators	Active treatment comparator	15	60
	Conservative treatment comparator	8	32
	No comparator	8	32
Study design	Randomized and non-randomized trials	1	4
	Observational studies	24	88
	Single arm studies or within-treatment comparisons^†^	2	8
Year of publication	2014	3	12
	2015	6	24
	2016	7	28
	2017	8	32
	2018	1	4
Total		25^‖^	100.0

*Studies may have multiple arms; percentages do not sum to 100

^†^Single-arm studies (or within-treatment comparisons) were eligible only for ablative therapies

^‖^The ROR approach missed a total of 26 eligible citations, which one was a companion study and one was the Fenton et al. review. The analysis is limited to the 25 primary studies

*Abbreviations*: *N* number, *NA* not applicable, *RCT* randomized controlled trial, *ROR* review of reviews

### Semi-automation approach: RobotAnalyst

The results of the semi-automation test using RobotAnalyst with a training set of random citations (Table 4) illustrated higher sensitivity for lower inclusion probability thresholds (100% for 30% inclusion probability threshold vs. 74% for 50% inclusion probability threshold), with no missed citations at a threshold of 30% and 12 missed citations at a threshold of 50%. However, at lower inclusion probability thresholds, specificity decreased (30% vs 55%) and the time saved was minimal (11 min for threshold 30%), as the algorithm at this threshold predicted that nearly all of the citations would be included (2168/2190 = 99%).

**Table 4:** Semi-automation test with RobotAnalyst using a training set of dually reviewed randomly selected citations with labels from title and abstract screening

	**Traditional database search** Total citations: 3128 Title-abstract screening: 148 includes/2980 excludes Full-text screening: 46 includes/3082 excludes		
	**Training set** Labeled citations: 938 (30%) Training set labels: TP (15), FP (32), TN (891) Unlabeled citations assigned inclusion prediction by ML algorithm: 2190		
	Inclusion prediction: 0.3	Inclusion prediction: 0.4	Inclusion prediction: 0.5
Predicted includes	2168	1970	1363
Predicted excludes	22	220	827
Sensitivity	100%	93%	74%
Specificity	30%	36%	55%
Missed citations	0	3	12
Burden	99%	93%	74%
Time savings (min)	11	110	413.5

*FP* false positive, *ML* machine learning, *TP* true positive, *TN* true positive

The semi-automation test using RobotAnalyst with the ROR training set showed similar trends of increased sensitivity at lower thresholds of inclusion probability (100% sensitivity for 30% inclusion probability threshold vs. 69% sensitivity for 50% inclusion probability threshold), though higher reviewer burden and minimal time saved (99% and 8 min, respectively) (Table 5). Compared to the ROR approach alone, the semi-automation approach using the ROR citations as the training set improved sensitivity and missed fewer citations even at the higher inclusion probability threshold. For example, for a threshold of 0.5, semi-automation using the ROR citations as a training set missed 18 citations compared with 26 citations.

**Table 5:** Semi-automation test with RobotAnalyst using a training set of dually reviewed citations from a review-of-review with labels from full-text screening

	**Traditional search** Total citations: 3181 Title-abstract screening: 201 includes/2980 excludes Full-text screening: 59 includes/3122 excludes			
	**Review of reviews** Total SR citations: 30 Total primary study citations: 95 Title-abstract screening: 76 includes/49 excludes Full-text screening 33 includes/43 excludes Number of citations not screened: 3030	**Training set: ROR citations** Labeled citations: 125 (4%) Training set labels: TP (33), FP (0), TN (92) Unlabeled citations assigned inclusion prediction by ML algorithm: 3056		
		Inclusion prediction: 0.3	Inclusion prediction: 0.4	Inclusion Prediction: 0.5
Predicted includes	NA	3040	2819	1166
Predicted excludes	NA	16	237	1890
Sensitivity	54%	100%	97%	69%
Specificity	100%	3%	10%	63%
Missed citations	26	0	2	18
Burden	4%	99%	93%	41%
Time savings (min)	1561	8	118.5	945

*FP* false positive, *ML* machine learning, *NA* not applicable, *TP* true positive, *TN* true positive

**Fig. 6:**
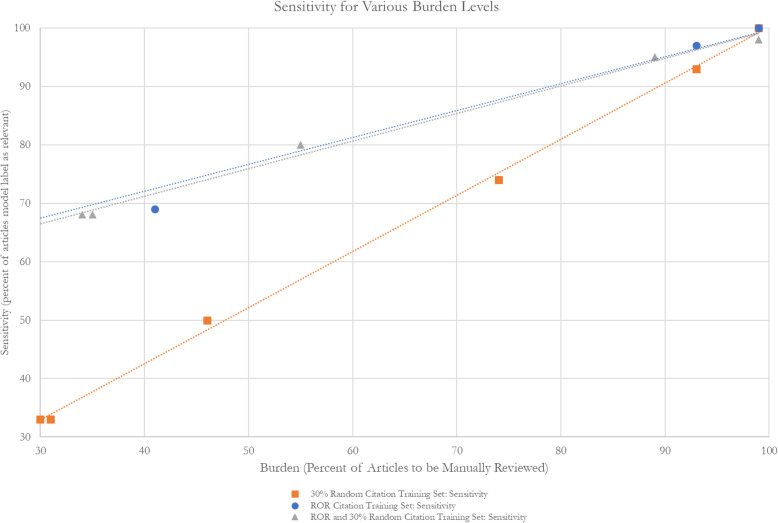
Burden and sensitivity outcomes for RobotAnalyst tests using alternative training sets

Compared to the training set of random citations (*n* = 938), the ROR training set was considerably smaller (*n* = 125), though demonstrated similar performance in terms of sensitivity and missed citations, as well as similar estimates of burden and time savings. Plotting sensitivity against burden shows that the semi-automation approach using a ROR training set performs slightly better than using a random dataset (Fig. 6). The third training set combined the ROR training set with a random set of citations, labeled with full-text decisions. The results of the semi-automation test for this training set were similar to the ROR test alone (Table 6, Fig. 6).

**Table 6:** Semi-automation test with RobotAnalyst using a training set of dually reviewed citations from a review-of-review and randomly selected citations with labels from full-text screening

	**Traditional search** Total citations: 3181 Title-abstract screening: 201 includes/2980 excludes Full-text screening: 59 includes/3122 excludes		
	**Training set: ROR citations + 30% random citations** Labeled citations: 1063 (33%) Training set labels: TP (40), FP (0), TN (1023) Unlabeled citations assigned inclusion prediction by ML algorithm: 2118		
	Inclusion prediction: 0.3	Inclusion prediction: 0.4	Inclusion prediction: 0.5
Predicted includes	2094	1765	676
Predicted excludes	24	353	1442
Sensitivity	98%	84%	80%
Specificity	34%	44%	79%
Missed citations	1	3	12
Burden	99%	89%	55%
Time savings (min)	12	177	721

*FP* false positive, *ML* machine learning, *TP* true positive, *TN* true positive

### Semi-automation approach: AbstrackR

**Fig. 7:**
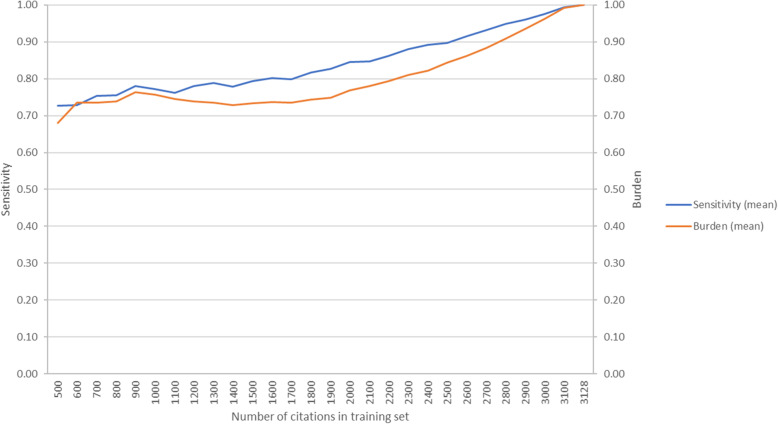
Sensitivity and burden using AbstrackR active learning for title-abstract screening

The semi-automation test of AbstrackR illustrated similar findings of increased sensitivity with larger training sets but increasing burden as well (Fig. 7, Appendix 5). For a training set of approximately 30% of the total citations, the mean sensitivity was 77% and the mean burden was 76%. Sensitivity did not exceed 90% until the training set includes 2500 of the 3128 citations.

## Discussion

In this study, we evaluated two strategies—a review of review approach and semi-automation—to update the most recent SRs on treatments of early prostate cancer. Review of reviews are a commonly employed strategy for rapid reviews [17] The ROR approach for treatments of early-stage prostate cancer had a poor sensitivity (0.56) and studies missed by the ROR approach tended to be of head-to-head comparisons of active treatments, observational studies, and more recent studies (as would be expected given the lag between literature searches and publication). Even though active comparators were part of the inclusion criteria in the majority of SRs in the ROR, 15 of the 25 missed citations (60%) included an active treatment comparison arm. Among missed studies with an active comparator, 11 studies were published in 2016 or later, suggesting comparative effectiveness studies may be missed due to the delay between SR search and publication. Approximately two-thirds of SRs captured by the ROR approach included trials as an eligible study design; only one missed citation was a trial. However, less than half of SRs included observational studies, and this study design accounted for nearly 90% of missed citations. Many evidence grading systems like GRADE have downgraded quality of observational studies [18] in the past, which can impact overall strength of evidence rating of an SR. Reviewers may decide to limit SR inclusion criteria to the highest quality research (i.e., trials) and exclude observational studies, which can also limit the search yield to a more manageable number of citations for a SR team. As a consequence of excluding observational study designs, quality of life and physical harms were among the most common missed outcomes in the ROR approach, which has implications for projects that focus on important patient-centered outcomes. Our results suggest careful consideration before employing a ROR approach to expedite the review process. Planning a priori to compare the inclusion criteria of a proposed ROR with those of retrieved SRs may help to proactively identify study characteristics for targeted primary literature searches.

The promise of ML to reduce reviewer burden of sometimes tedious SR steps like title and abstract screening has yet to be realized [10, 19]. Many prior studies have retrospectively evaluated the performance of ML tools, with knowledge of the final inclusion and exclusion decisions, which reviewers cannot know prospectively. In this study, we simulated three possible prospective uses of semi-automation: incorporating ML during title-abstract screening, adding ML to a ROR, and applying ML after conducting a ROR and a partial title-abstract and full-text review. By using a training set of random citations with labels determined by title-abstract screening, we simulated incorporation of ML at the title-abstract stage, where many citations labeled as included will go on to be excluded after full-text screening. In this SR update of early prostate cancer treatments, 148 abstracts from the database search were included after title and abstract screening and only 46 (31%) were included in the final SR update. Both tests of RobotAnalyst and AbstrackR using random citations with labels from title and abstract screening demonstrated acceptable sensitivity only at the high levels of burden that would not effectively reduce the workload compared to a traditional review. Our findings for AbstrackR tests mirrored RobotAnalyst tests in that increasing sensitivity was associated with increased reviewer burden. Unlike previous AbstrackR simulations, where the mean sensitivity curve rises quickly and flattens out at a high sensitivity at lower levels of burden, the sensitivity and burden curves had similar slopes [11]. This may have been due to this review having a small proportion of included studies (4%).

For semi-automation tests using the ROR training set and ROR with partial full-text review, we invested additional effort to create training sets for RobotAnalyst that only included labels determined after dual full-text review. We observed improved sensitivity of the ML-enhanced process for the same workload using a much smaller training set of curated citations (*n* = 125) compared with a training set of random citations (*n* = 938). (Figure 6) Removing false-positive labels applied at the title-abstract screening stage may reduce “noise” in the training set and more effectively train the ML algorithm [15]. Adding roughly a third of the search results with full-text inclusion labels to the ROR training set did not improve sensitivity much over the smaller training set. Prior studies have suggested that ML algorithms may perform better with training sets that are more balanced and less well in collections with a smaller proportion of includes [15, 16].

Our results suggest that the increased effort to create a more curated training set may reduce downstream burden; however, there remain several questions on how to include ML in SR processes. ML algorithms predict the probability of inclusion for a given citation, but it is unclear if and when human reviewers can safely stop screening without missing relevant studies. Even if a citation has a low inclusion probability, it may still be relevant [13, 20]. Even if a reviewer screens all citations using ML predictions to prioritize more relevant citations first, there is a risk of user complacency, agreeing with the prediction rather than screening randomly presented abstracts independently free of bias [13]. The content of the SR may also affect performance of a semi-automation approach. Our review included a breadth of interventions and study designs, including observational studies. A prior study evaluating ML in multiple SRs found that machine learning supported screening had improved sensitivity for SRs including only randomized controlled trails versus studies including observational studies like case series [16]. Another study found poorer sensitivity of semi-automation approaches for SRs including multiple study populations, such as young adults and adults [15].

Strengths of this study include evaluation of the ROR approach, a commonly used strategy to expedite the SR process, and simulation of possible prospective uses of semi-automation, particularly using the results of the ROR as a training set for semi-automated screening. Prior studies have suggested using the original SR as the training set for an update [21], though an SR update may have a different set of PICOTS than the prior review due to shifts in the population of interest, greater availability of diagnostic tools like biomarkers, advances in treatment options, and greater interest in patient-centered outcomes. Citations from a ROR train the machine learning algorithm with currently relevant data.

Limitations of the study include using a training set of random citations that were not truly random as we included at least 5% of citations that would have been included after title-abstract screening to ensure that the ML algorithm would be exposed to citations in the minority class. With the AbstrackR test, we had the capacity to perform 50 random training sets for multiple sizes and found comparable sensitivity and burden for a similarly sized training set used for the RobotAnalyst test. The time savings estimate may be lower than we found as abstracts screened earlier in title and abstract screening may require longer review than those screened towards the end. For both approaches—ROR and semi-automation, we must consider the generalizability of our results to other content areas and research questions. There is a risk of incorporation bias because the ROR is a subset of the gold standard of a traditional review, though the direction of this bias is to inflate sensitivity and specificity. Even with possible incorporation bias, the sensitivity and specificity of the ROR was poor. Finally, we used the traditional SR approach of dual abstract screening as the reference against which more expedient approaches of ROR and ML were compared. However, independent, dual review of abstracts does not preclude screening errors [22].

In conclusion, two approaches to rapidly update SRs—review-of-reviews and semi-automation—failed to demonstrate reduced workload burden while maintaining an acceptable level of sensitivity. We suggest careful evaluation of the ROR approach through comparison of inclusion criteria and targeted searches to fill evidence gaps as well as further research of semi-automation use, including more study of highly curated training sets, stopping rules for human screening, ML at the search stage to reduce yields, and prospective use of ML to reduce human burden in systematic evidence review.

## Supplementary information

**Additional file 1: Appendix Table 1.** PubMed search string and yield (11/27/2019). **Appendix Table 2.** Cochrane search string and yield (11/27/2019). **Appendix Table 3.** AHRQ Evidence Reports and Technology Assessments Search String and Yield (1/7/2019). Table 4. Drug Effectiveness Review Project Drug Class Reviews Search String and Yield (1/7/2019). Table 5. National Guidelines Search String and Yield (1/7/2019). Table 6. ClinicalTrials.gov Search String and Yield (1/10/2019). Table 7. HSRPproj Search String and Yield (1/10/2019). Table 8. PCORI Portfolio Search String and Yield (1/10/2019). Appendix 2: Inclusion and Exclusion Criteria. Appendix 3. Outcomes for Machine Learning Algorithm Only. Table 3A. Semi-automation test with RobotAnalyst using a Training Set of Randomly-Selected Dually-Reviewed Citations with Labels from Title and Abstract Screening: Outcome Metrics for Machine Learning Algorithm Only. Table 3B. Semi-automation test with RobotAnalyst using a Training Set of Dually-Reviewed Citations from a Review-of-Review with Labels from Full Text Screening: Outcome Metrics for Machine Learning Algorithm Only. Table 3C. Semi-automation test with RobotAnalyst using a Training Set of Dually-Reviewed Citations from a Review-of-Review and Randomly-Selected Citations with Labels from Full Text Screening: Outcome Metrics for Machine Learning Algorithm Only. Appendix 4. Cohort Studies Missed by Review of Reviews Approach. Appendix 5: Table AbstrackR Outcomes for Each Training Set.

## Data Availability

The datasets during and/or analyzed during the current study available from the corresponding author on reasonable request.
