# Supplementary material for: Comparison of a traditional systematic review approach with review-of-reviews and semi-automation as strategies to update the evidence
Source: Syst Rev. 2020 Oct 19;9:243. doi: 10.1186/s13643-020-01450-2 (PMC7574591; doi:10.1186/s13643-020-01450-2)
Supplement: Supplementary file 1 — Additional file 1: Appendix Table 1. PubMed search string and yield (11/27/2019). Appendix Table 2. Cochrane search string and yield (11/27/2019). Appendix Table 3. AHRQ Evidence Reports and Technology Assessments Search String and Yield (1/7/2019). Table 4. Drug Effectiveness Review Project Drug Class Reviews Search String and Yield (1/7/2019). Table 5. National Guidelines Search String and Yield (1/7/2019). Table 6. ClinicalTrials.gov Search String and Yield (1/10/2019). Table 7. HSRPproj Search String and Yield (1/10/2019). Table 8. PCORI Portfolio Search String and Yield (1/10/2019). Appendix 2: Inclusion and Exclusion Criteria. Appendix 3. Outcomes for Machine Learning Algorithm Only. Table 3A. Semi-automation test with RobotAnalyst using a Training Set of Randomly-Selected Dually-Reviewed Citations with Labels from Title and Abstract Screening: Outcome Metrics for Machine Learning Algorithm Only. Table 3B. Semi-automation test with RobotAnalyst using a Training Set of Dually-Reviewed Citations from a Review-of-Review with Labels from Full Text Screening: Outcome Metrics for Machine Learning Algorithm Only. Table 3C. Semi-automation test with RobotAnalyst using a Training Set of Dually-Reviewed Citations from a Review-of-Review and Randomly-Selected Citations with Labels from Full Text Screening: Outcome Metrics for Machine Learning Algorithm Only. Appendix 4. Cohort Studies Missed by Review of Reviews Approach. Appendix 5: Table AbstrackR Outcomes for Each Training Set. [file 13643_2020_1450_MOESM1_ESM.docx]

Appendix 1: Searches

Appendix Table 1. PubMed search string and yield (11/27/2019)

| Search | Query | Items Found |
| --- | --- | --- |
| #1 | ("Prostate"[Mesh]) OR "Prostatic Neoplasms"[Mesh] | 135981 |
| #2 | (cancer*[tiab] or carcinoma*[tiab] or neoplasm*[tiab]) | 2096820 |
| #3 | prostat*[tiab] | 198788 |
| #4 | (#2 and #3) | 138662 |
| #5 | ("prostate cancer"[tiab] OR "prostatic neoplasm*"[tiab]) | 102968 |
| #6 | (#1 or #4 or #5) | 176589 |
| #7 | ("watchful waiting"[tiab] OR "active surveillance"[tiab] OR LRP[tiab] OR RLRP[tiab] OR prostatectom*[tiab] OR radiotherap*[tiab] OR EBRT[tiab] OR IMRT[tiab] OR proton[tiab] OR (intensity[tiab] AND modulated[tiab] AND therap*[tiab]) OR brachytherap*[tiab] OR curietherap*[tiab] OR cryosurger*[tiab] OR cryotherap*[tiab] OR cryoablat*[tiab] OR Cyberknife[tiab] OR freezing[tiab] OR HIFU[tiab] OR (high[tiab] AND intensity[tiab] AND focused[tiab] AND ultrasound*[tiab])) | 356272 |
| #8 | ("Watchful Waiting"[Mesh] OR "Brachytherapy"[Mesh] OR "Cryosurgery"[Mesh] OR "Cryotherapy"[Mesh] OR "Freezing"[Mesh] OR "High-Intensity Focused Ultrasound Ablation"[Mesh] OR "Prostatectomy"[Mesh] OR "Radiotherapy"[Mesh]) | 260047 |
| #9 | "therapy" [Subheading] OR "Treatment Outcome"[Mesh] OR "Therapeutics"[Mesh] | 8335082 |
| #10 | (#7 or #8 or #9) | 8493210 |
| #11 | (("Early Diagnosis"[Mesh] OR early stage[tw]) or ("Early Diagnosis"[tiab] OR "early stage"[tiab])) | [181994](https://www.ncbi.nlm.nih.gov/pubmed/?cmd=HistorySearch&querykey=48) |
| #12 | "Early Detection of Cancer"[Mesh] OR "Early Medical Intervention"[Mesh] | [21950](https://www.ncbi.nlm.nih.gov/pubmed/?cmd=HistorySearch&querykey=47) |
| #13 | ("early stage*"[tiab] or "early detection"[tiab] or "localized"[tiab] or "localised"[tiab] or "early intervention"[tiab]) | [408919](https://www.ncbi.nlm.nih.gov/pubmed/?cmd=HistorySearch&querykey=108) |
| #14 | t1[tiab] or t2[tiab] or "stage 1"[tiab] or "stage one"[tiab] or "nonmetastatic"[tiab] or "non-metastatic"[tiab] | 144703 |
| #15 | #11 or #12 or #13 or #14 | [639164](https://www.ncbi.nlm.nih.gov/pubmed/?cmd=HistorySearch&querykey=109) |
| #16 | (#6 and #10 and #15) | [15093](https://www.ncbi.nlm.nih.gov/pubmed/?cmd=HistorySearch&querykey=111) |
| #17 | (#6 and #10 and #15) Filters: Publication date from 2014/01/01 | [3978](https://www.ncbi.nlm.nih.gov/pubmed/?cmd=HistorySearch&querykey=112) |
| #17 | (#6 and #10 and #15) Filters: Publication date from 2014/01/01; Humans | [3128](https://www.ncbi.nlm.nih.gov/pubmed/?cmd=HistorySearch&querykey=112) |

Appendix Table 2. Cochrane search string and yield (11/27/2019)

| Search | Query | Yield |
| --- | --- | --- |
| 1 | Prostate cancer prostat* AND (neoplasm* OR cancer* OR carcinoma*) | 12007 |
| 2 | Treatment options “watchful waiting” OR “active surveillance” OR LRP OR RLRP OR prostatectom* OR radiotherap* OR EBRT OR IMRT OR proton OR (intensity AND modulated AND therap*) OR brachytherap* OR curietherap* OR cryosurger* OR cryotherap* OR cryoablat* OR Cyberknife OR freezing OR HIFU OR (high AND intensity AND focused AND ultrasound*) | 35651 |
| 3 | Combine sets 1 AND 2 | 44147 |
| 4 | Limit 3 to: Publication date from 2014 to 11/2018 | 57 |

Appendix Table 3. AHRQ Evidence Reports and Technology Assessments Search String and Yield (1/7/2019)

| Search | Query | Items Found |
| --- | --- | --- |
| #1 | “Prostate Cancer”; Filters: Publication date from 2014/01/01 | 11 |

Abbreviation: AHRQ, Agency for Healthcare Research and Quality

Table 4. Drug Effectiveness Review Project Drug Class Reviews Search String and Yield (1/7/2019)

| Search | Query | Items Found |
| --- | --- | --- |
| #1 | Ctrl+F “prostate” | 0 |
| #2 | Ctrl+F “cancer” | 0 |

Table 5. National Guidelines Search String and Yield (1/7/2019)

| Search | Query | Items Found |
| --- | --- | --- |
| #1 | “Prostate Cancer”; Filters: Publication date from 2014/01/01 | 12 |

Table 6. ClinicalTrials.gov Search String and Yield (1/10/2019)

| Search | Query | Items Found |
| --- | --- | --- |
| #1 | “Localized Prostate Cancer” AND “Treatment”; Filters: Primary completion from 2014/01/01; Recruiting, not yet recruiting, active not recruiting, or enrolling by invitation | 29 |

Table 7. HSRPproj Search String and Yield (1/10/2019)

| Search | Query | Items Found |
| --- | --- | --- |
| #1 | “Localized Prostate Cancer” AND “Treatment”; Filters: Ongoing | 3 |

Table 8. PCORI Portfolio Search String and Yield (1/10/2019)

| Search | Query | Items Found |
| --- | --- | --- |
| #1 | Filters: Cancer; Prostate cancer; Ongoing or PCORI peer review | 9 |

Abbreviation: PCORI, Patient-Centered Outcomes Research Institute

Appendix 2: Inclusion and Exclusion Criteria

| Criterion | Inclusion | Exclusion |
| --- | --- | --- |
| Population | - Men with clinically localized prostate cancer (T1 or T2 disease) - Studies with mixed populations that include ≤10% T3 disease - Studies that do not report T-stage but describe the population as having clinically localized cancer | - Men with advanced prostate cancer (T3 disease or higher, positive lymph nodes or distant metastases), refractory or recurrent prostate cancer, or small cell prostate carcinoma - Studies with mixed populations that have >10% with T3 disease and no stratification of results - Studies that do not report T-stage and the population is described as having high risk prostate cancer without specifying if clinically localized |
| Interventions | - Active surveillance - Surgery - Radical prostatectomy (RP) (robotic, laparoscopic, open) - Radiation therapy (RT) - External-beam radiation therapy (EBRT) - Low-dose rate brachytherapy (BT) - High-dose rate BT - Stereotactic body radiation therapy (SBRT) - Photon therapy - Proton Therapy - Ablative/focal therapy - Cryosurgery - High-intensity focused ultrasonography (HIFU) - Focal ablative therapies (photodynamic therapy, MRI-TULSA, focal irreversible electroporation) - Combination therapy - RT + hormonal therapy (ADT) - EBRT + BT | - Treatments for advanced prostate cancer (bilateral orchiectomy, chemotherapy, cancer vaccines) - Primary androgen deprivation therapy (ADT) - RP combined with neo-ADT |
| Comparator | - Watchful waiting or no treatment - Active comparators listed above for active surveillance, surgery, RT, or combination therapies - No comparison for harms of focal therapy | - No comparison for benefits - No comparison for harms of active surveillance, surgery, RT, or combination therapies - Head-to-head comparisons of different types of RT (including RT vs RT+ADT) - Head-to-head comparisons of different types of surgery |
| Outcomes | - Benefits: - All-cause mortality - Prostate cancer-specific mortality - Metastatic disease (defined as M1) - Harms: - Physical harms of treatment, as measured by validated tools (EPIC, UCLA-PCI) - Erectile dysfunction - Urinary incontinence - Bowel dysfunction | - Outcomes other than those specified - Regional disease progression (N1) - Biochemical progression - Hormone levels - Health care costs |

(continued)

Appendix 2: Inclusion and Exclusion Criteria (continued)

| Criterion | Inclusion | Exclusion |
| --- | --- | --- |
| Outcomes (continued) | - Procedure complications - Biopsy complications (infection, hematuria, pain) - Surgical complications (perioperative mortality, bleeding, thromboembolic disease, rectal lesions, urinary fistulae) - Post-procedure hospitalization - Medication adverse effects - Hot flashes - Gynecomastia - Hepatitis - Osteoporosis - Cardiac risk - Cognitive function - Quality of life - Disease specific (Expanded Prostate Cancer Index Composite) - Psychological harms, as measured by validated tools - Depression - Anxiety - Procedure burden   Decision regret |  |
| Timing | Published in 2014 or later | Published before 2014 |
| Study design | - Systematic reviews (SRs) individual studies to identify randomized and nonrandomized controlled trials for benefits* - SRs with quality ratings of individual studies to identify randomized and nonrandomized controlled trials and observational studies for harms - Randomized and nonrandomized controlled trials and observational cohorts for benefits and harms - Case-control studies for harms - Case-series/single-arm studies for benefits and harms of focal therapy | - Case studies - Case-control studies for benefits - Case series/single-arm studies for benefits or harms of active surveillance, surgery, RT, or combination therapies |
| Other | English language | Non-English |

*: We expanded this criterion from SRs with quality ratings to all SRs on further review

**Appendix 3. Outcomes for Machine Learning Algorithm Only**

In this appendix, we present outcomes in Box 1 calculated using predictions from the machine learning algorithm alone, not including training set decisions assigned by human reviewers.

*Figure. 2X2 Matrix to calculate outcomes for machine learning algorithm only*

|  |  | **Human consensus decision** | | **Total** |
| --- | --- | --- | --- | --- |
|  |  | **Include** | **Exclude** |  |
| **ML algorithm only** | **Include** | a (TP) | b (FP) | a + b |
|  | **Exclude** | c (FN) | d (TN) | c + d |
| **Total** | | a + c | b + d | a + b + c + d |

Superscript T= training set, superscript U= unlabeled, FP= false positive, ML=machine learning, TP= true positive, TN= true positive

**Table 3A. Semi-automation test with RobotAnalyst using a Training Set of Randomly-Selected Dually-Reviewed Citations with Labels from Title and Abstract Screening: Outcome Metrics for Machine Learning Algorithm Only**

|  | **Traditional Database Search**  Total Citations: 3128  Title-Abstract Screening: 148 includes / 2980 excludes  Full Text Screening: 46 includes/ 3082 excludes | | |
| --- | --- | --- | --- |
|  | **Training Set**  Labelled citations: 938 (30%)  Training Set Labels: TP (15), FP (32), TN (891)  Unlabelled citations assigned inclusion prediction by ML algorithm: 2190 | | |
|  | Inclusion Prediction: 0.3 | Inclusion Prediction: 0.4 | Inclusion Prediction: 0.5 |
| Predicted Includes | 2168 | 1970 | 1363 |
| Predicted Excludes | 22 | 220 | 827 |
|  |  |  |  |
| Sensitivity | 100% | 90% | 61% |
| Specificity | 1% | 10% | 38% |
| Missed Citations | 0 | 3 | 12 |
| Burden | 69% | 63% | 44% |
| Time Savings (minutes) | 11 | 110 | 413.5 |

**Table 3B. Semi-automation test with RobotAnalyst using a Training Set of Dually-Reviewed Citations from a Review-of-Review with Labels from Full Text Screening: Outcome Metrics for Machine Learning Algorithm Only**

| Traditional Review Approach | **Traditional Search**  Total Citations: 3181  Title-Abstract Screening: 201 includes / 2980 excludes  Full Text Screening: 59 includes/ 3122 excludes | | |
| --- | --- | --- | --- |
| Expedited Review Approach | **Training Set: ROR citations**  Labelled citations: 125 (4%)  Training Set Labels: TP (33), FP (0), TN (92)  Unlabelled citations assigned inclusion prediction by ML algorithm: 3056 | | |
|  | Inclusion Prediction: 0.3 | Inclusion Prediction: 0.4 | Inclusion Prediction: 0.5 |
| Predicted Includes | 3040 | 2819 | 1166 |
| Predicted Excludes | 16 | 237 | 1890 |
|  |  |  |  |
| Sensitivity | 100% | 92% | 31% |
| Specificity | 1% | 8% | 62% |
| Missed Citations | 0 | 2 | 18 |
| Burden | 96% | 89% | 37% |
| Time Savings (minutes) | 8 | 118.5 | 945 |

FP= false positive, ML=machine learning, TP= true positive, TN= true positive

**Table 3C. Semi-automation test with RobotAnalyst using a Training Set of Dually-Reviewed Citations from a Review-of-Review and Randomly-Selected Citations with Labels from Full Text Screening: Outcome Metrics for Machine Learning Algorithm Only**

| Traditional Review Approach | **Traditional Search**  Total Citations: 3181  Title-Abstract Screening: 201 includes / 2980 excludes  Full Text Screening: 59 includes/ 3122 excludes | | |
| --- | --- | --- | --- |
| Expedited Review Approach | **Training Set: ROR citations + 30% Random Citations**  Labelled citations: 1063 (33%)  Training Set Labels: TP (40), FP (0), TN (1023)  Unlabelled citations assigned inclusion prediction by ML algorithm: 2118 | | |
|  | Inclusion Prediction: 0.3 | Inclusion Prediction: 0.4 | Inclusion Prediction: 0.5 |
| Predicted Includes | 2094 | 1764 | 676 |
| Predicted Excludes | 24 | 353 | 1442 |
|  |  |  |  |
| Sensitivity | 95% | 84% | 37% |
| Specificity | 1% | 17% | 68% |
| Missed Citations | 1 | 3 | 12 |
| Burden | 66% | 55% | 21% |

FP= false positive, ML=machine learning, TP= true positive, TN= true positive

Appendix 4. Cohort Studies Missed by Review of Reviews Approach

| Author, Year | Title | N | RT | RT | Ablative | AS | WW | Outcome |
| --- | --- | --- | --- | --- | --- | --- | --- | --- |
| C. Acar et al., 2014^17^ | Quality of life in patients with low-risk prostate cancer. A comparative retrospective study: brachytherapy versus robot-assisted laparoscopic prostatectomy versus active surveillance | 144 | x | x |  | x |  | QOL |
| P. Ravi et al., 2014^18^ | Mental health outcomes in elderly men with prostate cancer | 50,856 | x | x |  |  | x | PSYCH |
| M. Hjalm-Eriksson et al., 2015^19^ | Long-term health-related quality of life after curative treatment for prostate cancer: a regional cross-sectional comparison of two standard treatment modalities | 347 | x | x |  |  |  | QOL |
| L. A. Hampson, et al., 2015^20^ | Impact of age on quality-of-life outcomes after treatment for localized prostate cancer | 9,522 | x | x |  | x | x | QOL |
| G. L. Lu-Yao, et al., 2015^21^ | Primary radiotherapy vs. conservative management for localized prostate cancer—a population-based study | 57,749 |  | x |  |  | x | BEN |
| P. M. Putora, et al., 2016^22^ | Erectile function following brachytherapy, external beam radiotherapy, or radical prostatectomy in prostate cancer patients | 478 | x | x |  |  |  | QOL |
| L. Weissbach, et al., 2016^23^ | HAROW: the first comprehensive prospective observational study comparing treatment options in localized prostate cancer | 3,169 | x | x |  | x | x | BEN |
| S. Carlsson, L. et al., 2016^24^ | Population-based study of long-term functional outcomes after prostate cancer treatment | 5,944 | x | x |  |  |  | QOL |
| P. V. Barbosa, et al., 2016^25^ | Overall survival in patients with localized prostate cancer in the US Veterans Health Administration: Is PIVOT generalizable? | 35,954 | x | x |  |  | x | BEN |
| C. J. Wallis, et al., 2016^16,26^ | Cardiovascular and Skeletal-related Events Following Localized Prostate Cancer Treatment: Role of Surgery, Radiotherapy, and Androgen Deprivation | 60,156 | x | x |  |  |  | MED |
| S. Albisinni, et al., 2017^27^ | Comparing high-intensity focal ultrasound hemiablation to robotic radical prostatectomy in the management of unilateral prostate cancer: a matched-pair analysis | 110 | x |  | x |  |  | BEN, QOL |
| J. W. Jang, et al., 2017^28^ | Long-term quality of life after definitive treatment for prostate cancer: patient-reported outcomes in the second posttreatment decade | 194 | x | x |  |  |  | QOL |
| R. M. Hoffman, et al., 2017^29^ | Treatment decision regret among long-term survivors of localized prostate cancer: results from the Prostate Cancer Outcomes Study | 934 | x | x |  |  | x | PSYCH |

(continued)

Appendix 4. Cohort Studies Missed by Review or Review Approach (continued)

| Author, Year | Title | N | RT | RT | Ablative | AS | WW | Outcome |
| --- | --- | --- | --- | --- | --- | --- | --- | --- |
| S. B. Williams, et al., 2017^30^ | Risk of hospitalisation after primary treatment for prostate cancer | 29,571 | x | x |  |  |  | PROC |
| S. B. Williams, et al., 2017^31^ | Discerning the survival advantage among patients with prostate cancer who undergo radical prostatectomy or radiotherapy: the limitations of cancer registry data | 34,473 | x | x |  |  |  | BEN |
| P. Blanchard, et al., 2018^32^ | Quality of life after brachytherapy or bilateral nerve-sparing robot-assisted radical prostatectomy for prostate cancer: a prospective cohort | 391 | x | x |  |  |  | QOL |

Abbreviations: AS, active surveillance, BEN, benefit outcomes, MED, medication harms, N, number; PIVOT, Prostate Cancer Intervention versus Observation Trial; PROC, procedural harms, PSYCH, psychological harms, QOL, quality of life harms, RP, radical prostatectomy, RT, radiation therapy, WW, watchful waiting/conservative management

**Appendix 5: Table AbstrackR Outcomes for Each Training Set**

| Number of labelled citations | % of labelled citations | Sensitivity (mean) | Burden (mean) |
| --- | --- | --- | --- |
| 500 | 16% | 0.73 | 0.68 |
| 600 | 19% | 0.73 | 0.74 |
| 700 | 22% | 0.75 | 0.74 |
| 800 | 26% | 0.75 | 0.74 |
| 900 | 29% | 0.78 | 0.76 |
| 1000 | 32% | 0.77 | 0.76 |
| 1100 | 35% | 0.76 | 0.75 |
| 1200 | 38% | 0.78 | 0.74 |
| 1300 | 42% | 0.79 | 0.74 |
| 1400 | 45% | 0.78 | 0.73 |
| 1500 | 48% | 0.79 | 0.73 |
| 1600 | 51% | 0.80 | 0.74 |
| 1700 | 54% | 0.80 | 0.74 |
| 1800 | 58% | 0.82 | 0.74 |
| 1900 | 61% | 0.83 | 0.75 |
| 2000 | 64% | 0.84 | 0.77 |
| 2100 | 67% | 0.85 | 0.78 |
| 2200 | 70% | 0.86 | 0.79 |
| 2300 | 74% | 0.88 | 0.81 |
| 2400 | 77% | 0.89 | 0.82 |
| 2500 | 80% | 0.90 | 0.84 |
| 2600 | 83% | 0.92 | 0.86 |
| 2700 | 86% | 0.93 | 0.88 |
| 2800 | 90% | 0.95 | 0.91 |
| 2900 | 93% | 0.96 | 0.94 |
| 3000 | 96% | 0.98 | 0.96 |
| 3100 | 99% | 0.99 | 0.99 |
| 3128 | 100% | 1.00 | 1.00 |
